# Supplementary material for: Time-resolved serial femtosecond crystallography reveals early structural changes in channelrhodopsin
Source: eLife. 2021 Mar 23;10:e62389. doi: 10.7554/eLife.62389 (PMC7987342; doi:10.7554/eLife.62389)
Supplement: Supplementary file 1. — Values in parenthesis are those of the highest resolution shell. [file elife-62389-supp1.docx]

|  | Dark | Dark2  (10 Hz) | | 1 μs | | 50 μs | | 250 μs | | 1 ms | | 4 ms |
| --- | --- | --- | --- | --- | --- | --- | --- | --- | --- | --- | --- | --- |
| **Data Collection** |  |  | |  | |  | |  | |  | |  |
| Wavelength (Å) | 1.77 | | | | | | | | | | | |
| No. of collected images | 660157 | 37513 | | 148290 | | 131721 | | 104777 | | 151127 | | 189568 |
| No. of hit images | 68058 | 5727 | | 18005 | | 22999 | | 21516 | | 23512 | | 28997 |
| No. of indexed images | 62218 | 5356 | | 16924 | | 21129 | | 16442 | | 22156 | | 24015 |
| Space group | *C*222_1_ | | | | | | | | | | | |
| Cell dimensions |  |  | |  | |  | |  | |  | |  |
| *a*, *b*, *c* (Å) | 61.8, 142.2, 94.7 | | | | | | | | | | | |
| α, β, γ (°) | 90, 90, 90 | | | | | | | | | | | |
| Resolution (Å) | 15-2.3  (2.34-2.30) | 15-2.5  (2.54-2.50) | | 15-2.5  (2.54-2.50) | | 15-2.5  (2.54-2.50) | | 15-2.5  (2.54-2.50) | | 15-2.5  (2.54-2.50) | | 15-2.5  (2.54-2.50) |
| *R*_split_ | 0.061 (0.60) | 0.21 (0.90) | | 0.11 (0.58) | | 0.095 (0.66) | | 0.13 (0.89) | | 0.096 (0.51) | | 0.095 (0.64) |
| *I* / σ*I* | 9.62 (1.55) | 3.68 (1.24) | | 6.42 (1.66) | | 7.16 (1.63) | | 5.38 (1.16) | | 7.42 (2.00) | | 6.94 (1.56) |
| Completeness (%) | 100 (100) | 100 (100) | | 100 (100) | | 100 (100) | | 100 (100) | | 100 (100) | | 100 (100) |
| Multiplicity | 553 (139) | 43.3 (20.7) | | 140 (60.2) | | 155 (66.7) | | 119 (39.2) | | 180 (83.8) | | 172 (63.4) |
| CC_1/2_ | 1.00 (0.84) | 0.94 (0.52) | | 0.98 (0.80) | | 0.99 (0.78) | | 0.98 (0.64) | | 0.99 (0.82) | | 0.99 (0.79) |
|  |  |  |  | |  | |  | |  | |  | |
| **Refinement** |  |  |  | |  | |  | |  | |  | |
| Resolution (Å) | 14.96 - 2.5 |  |  | |  | |  | |  | |  | |
| *R*_work_ / *R*_free_ | 0.1847 / 0.2397 |  |  | |  | |  | |  | |  | |
| No. atoms |  |  |  | |  | |  | |  | |  | |
| Protein | 2317 |  |  | |  | |  | |  | |  | |
| Ligand/ion | 174 |  |  | |  | |  | |  | |  | |
| Water | 38 |  |  | |  | |  | |  | |  | |
| *B*-factors (A^2^) |  |  |  | |  | |  | |  | |  | |
| Protein | 70.64 |  |  | |  | |  | |  | |  | |
| Ligand/ion | 113.29 |  |  | |  | |  | |  | |  | |
| Water | 66.42 |  |  | |  | |  | |  | |  | |
| R.m.s. deviations |  |  |  | |  | |  | |  | |  | |
| Bond lengths (Å) | 0.0083 |  |  | |  | |  | |  | |  | |
| Bond angles (°) | 1.3929 |  |  | |  | |  | |  | |  | |
| Ramachandran plot |  |  |  | |  | |  | |  | |  | |
| Favored (%) | 97.93 |  |  | |  | |  | |  | |  | |
| Allowed (%) | 2.07 |  |  | |  | |  | |  | |  | |
| Outlier (%) | 0.00 |  |  | |  | |  | |  | |  | |
