# Supplementary material for: Time-resolved serial femtosecond crystallography reveals early structural changes in channelrhodopsin
Source: eLife. 2021 Mar 23;10:e62389. doi: 10.7554/eLife.62389 (PMC7987342; doi:10.7554/eLife.62389)
Supplement: Supplementary file 2. — Because of the large variance of the extrapolated structure factor amplitude, the R values tend to be worse. [file elife-62389-supp2.docx]

|  | 1 μs | 50 μs | | 250 μs | | 1 ms | | 4 ms | |  |
| --- | --- | --- | --- | --- | --- | --- | --- | --- | --- | --- |
| Activation ratio | 0.10 | | 0.15 | | 0.12 | | 0.12 | | 0.12 | |
| *F*_Extra_/σ(*F*_Extra_) | 1.67 | | 2.49 | | 1.74 | | 2.17 | | 2.14 | |
| *F*_Extra_ > 0 (%) | 74.5 | | 81.0 | | 81.3 | | 75.9 | | 81.4 | |
| **Refinement** |  | |  | |  | |  | |  | |
| Resolution (Å) | 14.96 - 2.5 | | 14.96 - 2.5 | | 14.96 - 2.5 | | 14.96 - 2.5 | | 14.96 - 2.5 | |
| *R*_work_ / *R*_free_ | 0.4448 /0.5148 | | 0.3844 /0.4471 | | 0.4467 /0.5141 | | 0.4106 /0.4634 | | 0.4048 /0.4869 | |
| No. atoms |  | |  | |  | |  | |  | |
| Protein | 2317 | | 2317 | | 2317 | | 2317 | | 2317 | |
| Ligand/ion | 174 | | 174 | | 174 | | 174 | | 174 | |
| Water | 38 | | 38 | | 38 | | 38 | | 38 | |
| *B*-factors (A^2^) |  | |  | |  | |  | |  | |
| Protein | 76.92 | | 64.14 | | 63.56 | | 74.27 | | 53.87 | |
| Ligand/ion | 73.57 | | 90.51 | | 77.77 | | 79.81 | | 65.51 | |
| Water | 45.06 | | 49.73 | | 37.55 | | 61.99 | | 28.59 | |
| R.m.s. deviations |  | |  | |  | |  | |  | |
| Bond lengths (Å) | 0.0116 | | 0.0079 | | 0.0100 | | 0.0090 | | 0.0081 | |
| Bond angles (°) | 1.5950 | | 1.4318 | | 1.4809 | | 1.5408 | | 1.4775 | |
| Ramachandran plot |  | |  | |  | |  | |  | |
| Favored (%) | 94.48 | | 94.83 | | 94.83 | | 94.83 | | 94.14 | |
| Allowed (%) | 8.68 | | 8.26 | | 9.09 | | 9.09 | | 9.09 | |
| Outlier (%) | 0.00 | | 0.00 | | 0.00 | | 0.00 | | 0.00 | |
